# Building a strong foundation: How pre-doctorate experience shapes doctoral student outcomes

**DOI:** 10.1371/journal.pone.0291448

**Published:** 2023-09-08

**Authors:** Svetlana Zhuchkova, Saule Bekova

**Affiliations:** 1 Center for Sociology of Higher Education, HSE University, Moscow, Russia; 2 Center for Institutional Analysis of Science & Education, European University at St. Petersburg, St. Petersburg, Russia; Instituto Tecnologico Autonomo de Mexico, MEXICO

## Abstract

The effectiveness of doctoral programs has been a major topic of interest for national policies, universities, and researchers for decades now. However, studies that try to identify factors associated with doctoral students’ success usually focus on characteristics measured during doctoral training, while the role of pre-doctorate characteristics remains underexplored. This research aims to fill this gap by examining whether and how various aspects of pre-doctorate experience–academic achievements, research experience related and unrelated to the dissertation topic, and teaching experience–contribute to the successful defense of the doctoral dissertation. Using data from a survey of the Russian doctoral programs’ graduates (N = 985) and regression analysis, we show that research experience related to the dissertation topic is the only pre-doctorate characteristic associated with the successful defense of the dissertation. At the same time, the effect of this type of research experience vanishes when controlling for support from the supervisor and department that students receive during their training. The results of the study can be used for designing criteria for doctoral students’ admission campaigns and introduction of integrated, or fast-track, doctoral programs, as well as to broaden our understanding of the relative importance of environmental vs. individual factors of doctoral students’ outcomes.

## Introduction

The effectiveness of doctoral programs has been a major topic of interest for national policies, universities, and researchers for decades now. These studies focused on factors that can improve doctoral completion and decrease time-to-degree. While the majority of these studies were devoted to institutional [1, 2], departmental [3, 4], and student-level [5, 6] factors measured during the training, there has been a limited number of research examining pre-doctorate characteristics [7].

Studies in this area usually focus either on unequal access to doctoral training or on the relationship between criteria used to assess prospective doctoral students and their outcomes. Both groups of studies showed that pre-doctorate characteristics are related to students’ completion and/or time-to-degree. Studies of inequality during the doctorate focus mostly on students’ background, e.g., race, gender, and family characteristics. These studies have shown that first-generation graduate students have specific challenges and lower chances of completing the program [8, 9], and students from underrepresented groups, such as black students, demonstrate lower completion rate [10]. Studies devoted to the relationship between admission criteria and students’ outcomes showed formal test results to be significant: undergraduate GPA and GRE are related to students’ time-to-degree [11], and GPA was also shown to be a predictor of program completion [12].

Another pre-doctorate characteristic significant for students outcomes is students previous research experience. Numerous studies have shown the important role of undergraduate research experience for PhD aspirations [13, 14], for boosting students confidence in their research and personal skills and understanding of how graduate schools work [15–17], and student chances to obtain a degree [18]. However, most of these studies assessed the effectiveness of a particular research program and covered primarily STEM field. In this study we will examine the wide range of research and learning undergraduate activities and their relationship with chances to obtain a degree based on cross-discipline survey.

Several studies also showed the significance of a master’s degree for PhD outcomes [7, 10]. Graduates of research universities are also more likely to finish graduate programs [9], and students whose supervisors conduct research in one of the strategic areas perform better in terms of publication activity [19].

The pre-doctoral students’ characteristics were also explored around the question of students’ initial motivation to pursue a doctoral degree and their expectations about it [20–24]. However, these studies are often case studies based on qualitative data with the main focus on developing typologies of studied concepts rather than on connecting them to graduation outcomes. Our study is based on a nationwide survey of graduates who finished doctoral programs in 2010–2021 in Russia. By exploring the role of pre-doctorate experience in doctoral completion, this study aims to provide insights into factors that can improve doctoral program effectiveness.

## National context

In 2020 Russian doctoral programs accepted 27 710 new doctoral students and awarded 3 834 new PhD holders [25]. Doctoral training in Russia is provided mostly by universities (87% of all doctoral students) while the remaining students are trained in research institutes (12%) and other organizations (1%; [26]). The majority of students are domestic; there are only 17% of international doctoral students in Russia, with many of them coming from post-Soviet states [26]. Most doctoral students study for free: in 2021, 59% of doctoral positions were funded by the state [26]. Although most positions are state-funded, the stipend provided to students is very low, leading many of them to work outside academia in non-research positions [27]. Universities cannot provide students with teaching or research workloads, contributing to the necessity of external employment [27].

In the past two decades doctoral training in Russia has faced a significant decline in the number of defenses: currently, around 10% of doctoral students defend their thesis during the expected degree date [25] and up to 45%–during next two years after graduation [28]. Around 40% of PhDs end up working in academia and 22% of those who didn’t defend their thesis work in the R & D area [28].

Various factors have contributed to this decline, with the selection system and quality of newly recruited PhD candidates being among the most influential [29]. The majority of organizations in Russia still utilize the Soviet-era formal selection system, which comprises three exams: major, foreign language, and philosophy [30]. The common usage of formal criteria leads to a growing share of students with non-academic motivation, irrelevant skills, and lower chances of success [31]. Although normative documents allow for a change in the selection system, only a limited number of organizations currently use alternative criteria emphasizing candidates’ previous learning and research experiences (2–25% depending on a specific procedure; [32]).

Studies of the role of pre-doctorate characteristics in students’ outcomes are rare in Russia. Few exceptions have shown that students with higher academic achievements at school and with more educated parents are more likely to pursue a doctoral degree [33]. Furthermore, students who had more than a 5-year gap before entering a doctoral program are more productive in publishing [34]. Finally, students that got their previous degrees in the same university have more chances to get a degree [27].

## Data

Data for this research were collected as part of a nationwide survey of graduates who finished doctoral programs in 2010–2021 in Russia. The survey was included in the project “Monitoring of Education Markets and Organizations” (MEMO) and supported by the Russian Ministry of Science and Higher Education (RMSHE). Data were collected online in June-July 2022.

Universities and research institutes were invited by the RMSHE to participate in the study and share a link to the questionnaire with graduates of their doctoral programs. Additionally, the link was placed on social media accounts of the same doctoral programs and the communities of their graduates. Participation in the survey was voluntary and anonymous. The authors had no access to information that could identify individual participants during or after data collection. The informed consent was integrated into the first screen of the online survey. Respondents were asked to tick the box if they agree to participate in the study. The study was approved by the Institutional Review Board of HSE University.

Overall, 1669 graduates participated in the survey. For our study, we selected only those participants who met the following criteria:

1) *they were enrolled in doctoral programs in 2013 and later*. Since then, doctoral education in Russia has been included in the system of higher education (instead of professional training). This criterion helped to ensure that the model and the legal framework of doctoral education remained the same for all graduates during their training.

2) *they studied at universities (not research institutes)*. Doctoral programs arranged in research institutes have specifics that may not be reflected in our data. Besides that, in the last 10 years, only a small part (10–13%) of all Russian doctoral students studying in research institutes [26]. In our sample, the number of such doctoral students was also not enough to conduct a reliable statistical analysis of this group of doctoral students. The results obtained in this study could not be generalized to this group of doctoral students.

As a result, 985 graduates from 119 universities were included in the consequent analysis. The anonymized data that were used in this research can be found in S1 Dataset. Fifty-five percent of this sample were females. More than half (56%) of the sample were enrolled in their doctoral programs in 2016 and later. Among the graduates, 29% were from social science, 27% from engineering and technology, 21% from mathematics and natural science, 15% from humanities, and 8% from education and pedagogy. Forty percent of the respondents studied at leading universities (universities that participated in specific excellence programs), 85% were full-time students, and 77% studied with a tuition-free form of financing. While all the respondents finished their doctoral programs and received a diploma, only 38% defended their dissertations.

## Materials

To describe their pre-doctorate experience, graduates answered the question:

Could you please remember which of the following activities you did before you were enrolled in your doctoral program?

1. *Participated in Russian or foreign research conferences*
2. *Conducted or participated in research related to your future dissertation*
3. *Conducted or participated in research* *not* *related to your future dissertation*
4. *Published papers on the topic related to your future dissertation*
5. *Published papers on the topic* *not* *related to your future dissertation*
6. *Participated in competitions for research papers*
7. *Took first*, *second*, *or third places in student olympiads*
8. *Received additional scholarships/funding for your academic or research activities*
9. *Received a diploma with honors in any of your previous higher education programs*
10. *Taught in a school or a college*
11. *Taught at a university*
12. *None of the above*

The majority of the presented options (papers, conferences, competitions, olympiads, scholarships, diploma with honors) correspond to the student’s achievements that universities in Russia usually take into account during their admission campaigns [32]. However, research experience is not always converted into such formal achievements. To take into account the practices beyond the limited scope of the performance indicators, we included some general options (“Conducted or participated in research…”). We also divided some options into two versions describing experiences related and unrelated to the topic of a student’s dissertations as many institutions distinguish these types of experiences and require applicants to have experience specifically related to their dissertations [32]. Finally, since a significant proportion of Russian students pursue a doctorate to advance their careers as instructors or teachers in universities or other educational organizations (48% in the study [35] and 43% in our sample), options that reflect teaching experience were also included in the question.

**Fig 1:**
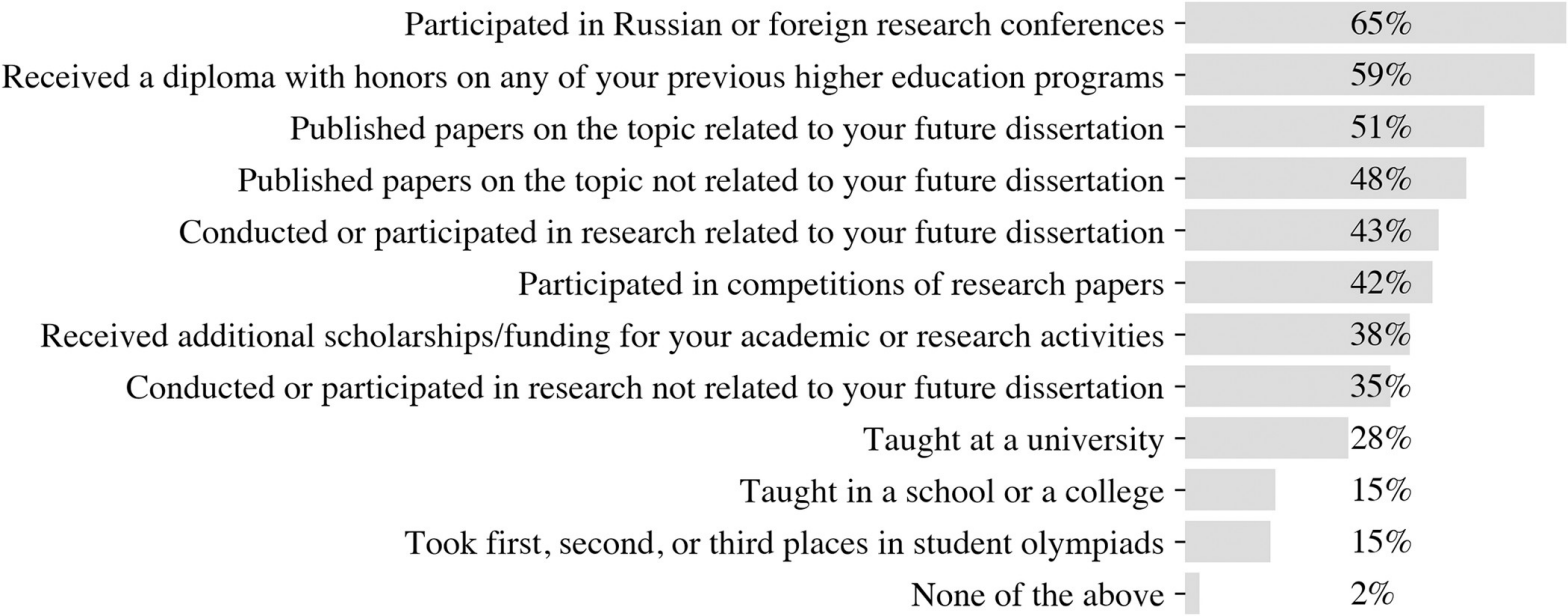
Prevalence of different forms of pre-doctorate experience. Question: “Could you please remember which of the following activities you did before you were enrolled in your doctoral program?” (N = 985).

In our sample, the most common form of pre-doctorate activities is visiting research conferences (65%; Fig 1). Winning olympiads and teaching experience are the least frequent activities students were engaged in before their doctorate (28% taught at university, 15% taught in school or college; 15% won olympiads). In general, more than a third of students conducted or participated in research related (43%) or unrelated (35%) to their future dissertation.

To explore associations between the pre-doctorate experience and the consequent defense of dissertation, we also used a set of control variables that may also be associated with a successful defense.

The first control variable is related to the students’ motives to pursue a doctoral degree. To get this variable, we used the following multiple-choice question:

What goals did you pursue when enrolling in a doctoral program?

1. *To receive a scientific degree*
2. *To receive a diploma without the defense of dissertation*
3. *To enhance my research skills*
4. *To enhance my teaching skills*
5. *To continue researching a topic I am interested in*
6. *To advance my career in academia*
7. *To advance my career outside academia*
8. *To go to a foreign university as part of a doctoral program*
9. *To get a job in this university/research institute*
10. *To get a postponement from army*
11. *To live in a student dormitory*

To include in the consequent analysis, this variable was recoded into a binary one that reflects the presence of non-academic motives to pursue a doctoral degree. Value 1 (“Had non-academic motives”) was assigned to the respondents who chose any of the following options: 2, 7, 10, or 11. If none of the mentioned options were chosen, value 0 (“Did not have non-academic motives”) was assigned to the respondent. In our sample, 34% of the graduates had such non-academic motives when enrolling in their doctoral programs.

The second control variable describes the presence and type of students’ employment during their doctoral training. Due to the low funding of doctoral education, combining work and study is extremely frequent in Russia with more than 90% of doctoral students working during their training [35]. However, the effects of the employment may vary depending on the place, position, etc., and may both increase and decrease the chances of successfully defending a dissertation [36]. In line with previous research on the role of employment at Russian doctoral programs [27], we distinguished four possible types of employment: not employed, employed outside the university, had a research position at the university, and had other position at the university. To get such a variable, we combined graduates’ answers to three questions: “Did you work during your doctoral training?”, “Did you work at your university during your doctoral training?”, and “What was your position at the job at your university during your doctoral training?” (options: teaching position, research position, administrative position, other position). In our sample, 8% of the graduates were not employed during their doctorates, 38% were employed outside their universities, 24% held research positions, and 30% held other positions in their universities.

The third and fourth control variables indicate the support that students received from their supervisors or departments during their doctoral training. In the study that reviewed different factors related to doctoral students’ attrition, a student-supervisor relationship was found to be the most frequent and influential factor examined in empirical research [37]. Supervisors are the main figures for doctoral students who not only advise on research problems and principles, but also become providers between students and the wider research community by engaging the former in different academic activities [38–40]. The department may also be a significant contributor to students’ outcomes as it may support students academically, socially, and organizationally [4], which is why it is frequently considered a primary agent for doctoral students’ socialization [40]. The indicators that reflected support from supervisors and departments were retrieved from the following, more general, question: “To what extent did you experience the following aspects during your doctoral training?” (scale: to a great extent, to a little extent, I did not experience this aspect). Among other possible statements, this question included options “Help and support from the supervisor” and “Help and support from the department”. To use these options in the analysis, we transformed them into binary variables by merging the categories “to a little extent” and “I did not experience this aspect” so that the eventual binary variables reflected only significant support from supervisors and departments (the initial category “to a great extent”). In our sample, 74% of graduates reported that they experienced significant support from their supervisors and 45% reported significant support from the department. Only these two variables in our analysis contained missing data which were treated using listwise deletion due to a small proportion of missingness (18 respondents had missing data in at least one of these variables).

## Methods

The analysis of the data was divided into two stages. At the first stage, we merged the initial indicators of the pre-doctorate experience into several components by means of the principal component analysis (PCA). Since these indicators were presented as binary variables, the PCA model was built on a tetrachoric correlation matrix using the *psych* R package [41]. We compared models with three, four, and five components to choose the final PCA model. All models were built using promax rotation as we expected the components of the pre-doctorate experience to be correlated. The final model was chosen as one with easily interpreted and contrast components. This model consisted of four components that accounted for 73% of the initial variance and were referred to as 1) academic achievements, 2) research experience related to the dissertation topic, 3) research experience not related to the dissertation topic, and 4) teaching experience. Factor loadings and communalities of the indicators are presented in Table 1.

**Table 1:** Factor loadings and communalities of the final PCA model.

Variable	PC1	PC2	PC3	PC4	Communality
Took first, second, or third places in student olympiads	0.799	0.024	-0.169	0.378	0.685
Received additional scholarships/funding for your academic or research activities	0.752	0.064	0.177	-0.061	0.720
Received a diploma with honors on any of your previous higher education programs	0.675	0.013	0.12	-0.077	0.531
Participated in competitions of research papers	0.569	0.223	0.188	0.115	0.615
Taught at a university	-0.498	0.333	0.292	0.372	0.621
Published papers on the topic related to your future dissertation	0.003	0.973	-0.169	-0.060	0.811
Conducted or participated in research related to your future dissertation	0.16	0.925	-0.198	-0.21	0.784
Participated in Russian or foreign research conferences	0.179	0.439	0.32	0.073	0.563
Published papers on the topic *not* related to your future dissertation	0.032	-0.203	0.985	-0.031	0.845
Conducted or participated in research *not* related to your future dissertation	0.131	-0.134	0.873	-0.124	0.729
Taught in a school or a college	0.225	-0.223	-0.125	0.96	0.816

PC stands for principal component. PC1 –academic achievements, PC2 –research experience related to the dissertation topic, PC3 –research experience not related to the dissertation topic, PC4 –teaching experience. The question used in the analysis: “Could you please remember which of the following activities you did before enrollment in your PhD program?” (multiple choice).

As a result of this stage of the analysis, four standardized variables were obtained that reflected various dimensions of the pre-doctorate experience. The bigger the value of each variable is, the more experienced a graduate was in this aspect before entering his or her doctoral program. Those graduates who have a minimum level for all the components correspond to those who chose the option “None of the above” when describing their pre-doctorate experience (therefore, this option was not included in the PCA model). The distribution of the most extracted components significantly differs between various groups of doctoral students (see Table 2). Male students demonstrate a higher level of academic achievements while female students are more experienced in teaching. Students of leading universities are distinguished by a higher level of all the components except teaching. Those who study full-time also reach more academic achievements and teach less before their doctoral training. Tuition-free students are characterized by a higher level of academic achievements and have more dissertation-related research experience. Finally, doctoral students in Humanities, as well as in Education and Pedagogy are more experienced in teaching than students in other fields.

**Table 2:** Mean values of the extracted components and their statistical differences.

Variable	Category	Academic achievements	Research experience related to the dissertation topic	Research experience not related to the dissertation topic	Teaching experience
Gender	Male	0.074	0.005	0.012	-0.112
	Female	-0.042	0.005	-0.007	0.085
	Test statistics	2.068 *	0.003	0.328	-3.273 ***
Leading university	No	-0.072	-0.051	-0.068	0.049
	Yes	0.133	0.089	0.107	-0.080
	Test statistics	-3.607 ***	-2.359 *	-2.956 **	2.098 *
Form of studying	Part-time	-0.324	-0.002	-0.075	0.359
	Full-time	0.070	0.006	0.016	-0.068
	Test statistics	-5.132 ***	-0.095	-1.118	4.611 ***
Form of financing	Tuition-based	-0.272	-0.132	-0.070	0.106
	Tuition-free	0.094	0.046	0.023	-0.035
	Test statistics	-5.613 ***	-2.691 **	-1.348	1.944
Field of study	Mathematics and natural science	0.070	0.057	-0.043	-0.169
	Engineering and technology	0.111	0.042	-0.065	-0.224
	Social science	-0.090	-0.070	0.129	-0.045
	Humanities	-0.034	-0.067	-0.087	0.369
	Education and pedagogy	-0.050	0.139	0.050	0.637
	Test statistics	2.245	1.420	2.276	21.260 ***

* p<0.05

** p<0.01

*** p<0.001

T-test statistics is presented for all variables except field of study. For field of study F-statistics is presented. The values of the standardized variables could be interpreted as follows: negative values mean that the value for this group is below the all-sample average, positive values mean that the value for this group is higher than the all-sample average. Zero represents the all-sample average.

At the second stage of the analysis, we explored associations between the extracted components of the pre-doctorate experience and the defense of the dissertation. Several models of binary logistic regression were built with the fact of dissertation defense as a dependent variable (the question “Have you defended your dissertation?”; 38% of the sample defended their dissertations) and different sets of independent variables. The first model focused solely on the effects of the pre-doctorate experience (four components extracted earlier). Then, in the next models, we consequently added the previously described control variables:

- presence of non-academic motives to pursue a doctorate (model 2),
- presence and type of employment during doctoral training (model 3),
- presence of significant support from supervisor (model 4) and department (model 5).

Additionally, we included the following control variables in all models: year of enrollment, form of studying, form of financing, type of university (leading or not), field of study, and gender.

## Results

The regression analysis shows that pre-doctorate experience, indeed, is associated with a successful defense of the dissertation, however, not all of its components matter, and not in all examined specifications it has a significant effect (see odds ratios in Table 3).

**Table 3:** Results of the logistic regression analysis (odds ratios).

Variable	Model				
	(1)	(2)	(3)	(4)	(5)
Intercept	1.32	1.68	2.66 *	1.16	1.07
Academic achievements	1.11	1.10	1.09	1.11	1.08
Research experience related to the dissertation topic	1.27 **	1.26 **	1.22 *	1.17	1.15
Research experience not related to the dissertation topic	0.97	0.99	0.99	1.00	1.00
Teaching experience	0.93	0.92	0.94	0.93	0.94
Non-academic motives		0.59 ***	0.61 **	0.61 **	0.61 **
Employment status (reference category–Not employed)					
Employment outside university			0.49 **	0.49 *	0.53 *
Research position at university			0.88	0.93	0.96
Another position at university			0.57 *	0.58	0.61
Significant support from supervisor				2.50 ***	2.11 ***
Significant support from department					1.55 **
Female	0.92	0.83	0.82	0.83	0.82
Leading university	1.10	1.07	1.04	1.17	1.19
Full-time	1.34	1.34	1.21	1.24	1.21
Tuition-free	0.84	0.85	0.88	0.88	0.95
Field of study (reference category–Mathematics and natural science)					
Engineering and technology	1.57 *	1.59 *	1.63 *	1.72 **	1.70 *
Social science	0.88	0.87	0.96	1.04	1.01
Humanities	1.37	1.32	1.48	1.49	1.51
Education and pedagogy	0.78	0.77	0.80	0.90	0.84
Year of enrollment	0.69 ***	0.69 ***	0.69 ***	0.69 ***	0.68 ***
Observations	985	985	985	973	967
R^2^ Tjur	0.105	0.116	0.129	0.156	0.164

* p<0.05

** p<0.01

*** p<0.001

Model 1 demonstrates that, among the components of the pre-doctorate experience, only research experience related to the dissertation topic is significantly associated with the successful defense of a dissertation. One standardized unit of this component increases the probability to defend a dissertation by 27%. Other components of the pre-doctorate experience used in our study, i.e., previous academic achievements, research experience not related to the dissertation topic, and teaching experience do not contribute significantly to the defense.

The identified effect of the research experience related to the dissertation topic remains significant in models 2 and 3 as well. Model 2 shows that the presence of non-academic motives reduces students’ chances to defend a dissertation (by 39%) but does not neutralize or change previously described effects of the pre-doctorate experience. Model 3 shows that different effects are provided by different types of employment. These effects partially reproduce findings obtained previously [27]: employment outside the university and holding an on-campus position different than a research one reduce students’ chances to defend a dissertation (by 51% and 44%, respectively) while the absence of employment and research position at university are associated with high chances to defend a dissertation (see Intercept in Model 3) and the effects of this types do not differ significantly. Similarly to the previous two models, research experience related to the dissertation topic still significantly increases the chances for defense.

Finally, the last two models stand out from others in regard to the effects of the pre-doctorate experience. These models show that, when controlling for support from the supervisor and department, the effect of research experience related to the dissertation topic becomes not big enough to be considered significant. The strongest effect is observed for support from the supervisor, receiving which increases the chances for successful defense by more than two times. Support from the department additionally increases these chances by 55%, all other things being equal.

## Discussion and conclusion

This study was aimed at exploring the role of various aspects of pre-doctorate experience in the consequent successful defense of dissertations by Russian doctoral students. We highlight two main findings that may contribute to the current literature on the factors associated with doctoral students’ success.

First, we demonstrate that, among different components of the pre-doctorate experience, only research experience related to the dissertation topic is associated with the students’ outcomes, increasing the chances of students to complete their dissertations. Other components, which include various academic achievements, general research experience, i.e., unrelated to the dissertation, and teaching experience, do not significantly contribute to the defense. This result supports previous findings on the importance of prior research experience for success at the doctoral level [7, 9, 16, 18]. This study enhances the existing literature by presenting a comprehensive list of indicators of pre-doctorate learning and research experience. Unlike previous studies that often assessed particular undergraduate research programs in STEM field, this study examined the role of wide list of pre-doctorate research activities based on the cross-discipline sample. Besides, in contrast to studies focused on formal characteristics such as scores of standardized tests [12], publications [19], master’s degree [10], this research covers indicators of both formal achievements and substantive aspects of research, educational, and professional experience.

Second, we show that the identified effect of the pre-doctorate research experience related to the dissertation topic disappears when controlling for the support received by doctoral students from their supervisors and departments. The vanishing significance of pre-doctoral experience may imply, among other things, that the support provided to students may have been a contributing factor to the previously established effect. Students with prior research experience might have built relationships with their future supervisors and departments that consequently resulted in a higher level of support from them. Alternatively, supervisors and departments may have paid more attention to the students who are widely experienced with their topics as they seem to be more prepared for successful research during their doctorate. This finding contributes to the more general discussion about the relative importance of environmental/institutional vs. individual factors of doctoral students’ success, which are rarely examined in a single study simultaneously [19, 42, 43]. The obtained result provides evidence that adequate support, including distributive support (from departments), may compensate for the lack of experience and unpreparedness of doctoral students and help them succeed in terms of the defense of a dissertation.

The results obtained in our research may be taken into account when designing criteria for admission campaigns at doctoral programs and thus may enhance the effectiveness of the selection process which in Russia is highly criticized by researchers [44, 45]. Our results explain the low effectiveness of the selection process in Russia by showing that the most frequent criteria that are taken into account by Russian universities–formal academic achievements, according to the study of Zhuchkova [32]–are not associated with successful defense in the future. And in contrast, those criteria that can reflect the only significant factor (research experience related to the dissertation topic), such as the quality of a project proposal, are the least widespread in Russian universities [32].

Besides that, the identified effect indicates the importance for supervisors and the research community to start working on a particular topic with potential doctoral students earlier–before the latter enter doctoral programs. At the institutional level, this can be achieved by the introduction of integrated, or fast-track, doctoral programs that imply that students begin their dissertation research at the level of master’s programs. Different modifications of such models are implemented in American, European, and Chinese universities [46], and some examples of the integrated tracks are currently being adopted by Russian universities as well [47].

The study has several limitations. First, we could encounter a survival bias as our data cover only those students who finished their doctoral programs. The effects of the pre-doctorate experience that we discovered for such students could differ from those of students who left their doctoral programs or were expelled during their studying. Second, although we emphasize the cross-discipline nature of our data, the small sample size does not allow us to explore the effects of the pre-doctorate experience in different fields of study separately. We expect that different components could matter for different fields as doctoral education has many discipline-related specifics, including inconsistent attrition and persistence rates for various fields of study [36]. Third, we use the fact of dissertation defense as our main dependent variable and take into account neither the quality of these dissertations nor the consequent trajectory of the graduates or their contribution to science and economy–the variables that could be more important for doctoral education evaluation and development, but which are unavailable in our data. Fourth, we rely on retrospective data as survey participants had to recall activities that they used to do a long time ago when answering the question about their pre-doctorate experience. This could affect the quality of our data given the natural restrictions of humans’ memory.

To overcome the abovementioned restrictions, we need longitudinal data of a bigger size that trace students’ experience before, during, and after the doctoral training. Such data would be more objective and reliable to answer our research questions in more detail, however, longitudinal data that cover nation-level samples are very rare and expensive. Only a few examples of studies that use longitudinal data about doctoral students exist (e.g., [2, 27, 48]), but they rely on administrative data and thus are limited to one or several institutions.

Finally, although we tried to account for different factors that could also be associated with a successful defense of the dissertation, our analysis still misses some important control variables such as the financial support of doctoral students during their training. However, in Russia practices of additional financial support for doctoral students are quite rare and usually assigned to those doctoral students who already demonstrated wide research experience and academic achievements. So, we may hypothesize that such a factor would be highly correlated with the components of the pre-doctorate experience and thus may become an additional explanation to the effects that we observed on our analysis. Nevertheless, further studies are needed to explore the set research questions in more detail.

## Supporting information

S1 Dataset Anonymized data used in the research. (XLSX)
